# Search-based multi-vulnerability testing of XML injections in web applications

**DOI:** 10.1007/s10664-019-09707-8

**Published:** 2019-04-13

**Authors:** Sadeeq Jan, Annibale Panichella, Andrea Arcuri, Lionel Briand

**Affiliations:** 1 SnT, University of Luxembourg, Luxembourg, Luxembourg; 2 Department of Computer Science & IT, University of Engineering & Technology, Peshawar, Pakistan; 3 Delft University of Technology, Delft, Netherlands; 4 Faculty of Technology, Kristiania University College, Oslo, Norway

**Keywords:** Security testing, Code injection vulnerabilities, Search-based software engineering

## Abstract

Modern web applications often interact with internal web services, which are not directly accessible to users. However, malicious user inputs can be used to exploit security vulnerabilities in web services through the application front-ends. Therefore, testing techniques have been proposed to reveal security flaws in the interactions with back-end web services, e.g., XML Injections (*XMLi*). Given a potentially malicious message between a web application and web services, search-based techniques have been used to find input data to mislead the web application into sending such a message, possibly compromising the target web service. However, state-of-the-art techniques focus on (search for) one single malicious message at a time.

Since, in practice, there can be many different kinds of malicious messages, with only a few of them which can possibly be generated by a given front-end, searching for one single message at a time is ineffective and may not scale. To overcome these limitations, we propose a novel co-evolutionary algorithm (COMIX) that is tailored to our problem and uncover multiple vulnerabilities at the same time. Our experiments show that COMIX outperforms a single-target search approach for *XMLi* and other multi-target search algorithms originally defined for white-box unit testing.

## Introduction

Web applications often rely on interactions with internal web services, e.g., SOAP (Curbera et al. 2002) and REST (Fielding 2000). This is a typical case for example in microservice architectures (Newman 2015). When web applications become too large and complex to develop and maintain, splitting them into smaller services helps to reduce their complexity. Despite being more flexible, scalable and maintainable, microservice architectures are characterized by a larger attacks surface due to increased communication complexity (Sharma and Gonzalez 2017). Indeed, in addition to every single microservice, hackers can exploit communication channels among microservices (e.g., front-end web applications and back-end web services) and try to compromise the entire system.

In the context of web applications, a major security concern is the validation and sanitization of user inputs (e.g., text strings in HTML input forms) which are checking for malicious content. Input validation discards user-supplied data if it does not conform to a specified rule or set of rules. On the other hand, input sanitization removes some special characters (e.g., <) from user inputs to prevent many kinds of possible attacks. These procedures are usually performed by front-end web applications that process and embed user inputs into messages (e.g., XML messages) for internal web services.

When input validation and sanitization procedures are not properly implemented, malicious inputs can be used to attack internal web services leading to different kinds of security attacks, such as XML injection (*XMLi*) and XSS attacks (Williams and Wichers 2013). Due to time pressures or lack of familiarity with security issues, such vulnerabilities are common in practice (Jan et al. 2015, 2016).

For these reasons, researchers have proposed various techniques (Liu and Tan 2008; Jan et al. 2017a; Kosuga et al. 2007) to test input validation and sanitization routines in web applications against different types of security attacks.

Recently, we proposed a black-box technique (Jan et al. 2017a, 2017b) based on genetic algorithms (GAs) to generate malicious user inputs that, once validated and processed by the front-ends, result in malicious XML messages potentially affecting internal web services. Given a malicious message *X* that could affect internal web services, search-based software testing techniques are then used to find user inputs to the front end (i.e., strings for web application form) that would lead to the generation of *X*. The search is guided by the edit distance (string (Jan et al. 2017a) or real-coded (Jan et al. 2017b) distance) between the message generated with the given user inputs and the target malicious message *X* (Jan et al. 2017a). If such user inputs are found, then the front-end is deemed vulnerable since it is not able to prevent the generation of *X*.

The main advantage of the aforementioned black-box approach is that it does not need to access the source code (of neither front-ends nor internal web services) and it can discover different types of vulnerabilities (Jan et al. 2017a). However, existing techniques focus on one single message at a time and, therefore, require to run GAs many times, once for each potential malicious message *X*. Since the number of messages can be large in practice when considering multiple types of attacks, searching for a single message/vulnerability at a time is inefficient (single-target approach) and not scalable to many large applications. First, not all target messages are feasible since the input validation would likely detect and filter out many malicious messages. Second, searching for malicious inputs related to some messages may be more difficult than others. Therefore, when the goal is to detect as many vulnerabilities as possible within time constraints, the order by which messages are selected for testing may impact the overall effectiveness (i.e., the number of detected vulnerabilities).

In this paper, we investigate different strategies targeting all malicious messages at the same time, which aim to overcome potential scalability challenges with the single-target approach. In the context of white-box unit testing, various search techniques (Panichella et al. 2015, 2017; Arcuri 2017) have been successfully used to cover multiple structural targets at the same time (e.g., branches). In our context, these techniques can be adapted and applied to detect *XMLi* attacks in front-end web applications. More specifically, in this paper, we investigate the performance of MOSA (Panichella et al. 2015) and MIO (Arcuri 2017), which are the most recent and effective techniques for white-box unit testing. To tailor it to our context, we adapt MOSA by developing a novel variant, which we call vMOSA. Moreover, we propose a novel search technique (COMIX), which is based on a cooperative, co-evolutionary search and is specifically designed for the *XMLi* testing problem. Finally, we investigate the usage of an alternative fitness function, which is much less expensive but possibly provides less guidance than the string edit distance commonly-used in search-based software testing (Jan et al. 2017a; Alshraideh and Bottaci 2006).

We evaluated these strategies by conducting an empirical study involving different versions of three web applications. Our results show that (i) all multi-target techniques outperform the single-target approach, and (ii) the novel co-evolutionary algorithm (COMIX) is significantly more effective and more efficient than both vMOSA and MIO, independently of the used fitness function. Finally, when the number of target messages increases, the fitness function we propose clearly helps all techniques to achieve better results.

The paper is organized as follows. Section 2 briefly describes *XMLi*, prior testing techniques for *XMLi*, the state-of-the-art multi-target techniques for white-box unit testing, and background information about co-evolutionary algorithms. Section 3 introduces our novel co-evolutionary algorithms and the proposed alternative fitness function. Sections 4 and 5 describe our empirical study and report our results, respectively. Section 6 discusses threats to validity while Section 7 summarizes related work. Finally, Section 8 concludes the paper.

## Background

This section briefly describes (i) *XMLi* attacks; (ii) search-based approaches for testing front-end web applications to detect these attacks; (iii) multi-target, search-based approaches used in white-box unit testing that we adapt to the context of *XMLi* vulnerability detection; and (iv) background information about co-evolutionary algorithms.

### XML Injection and Testing Context

**Fig. 1:**
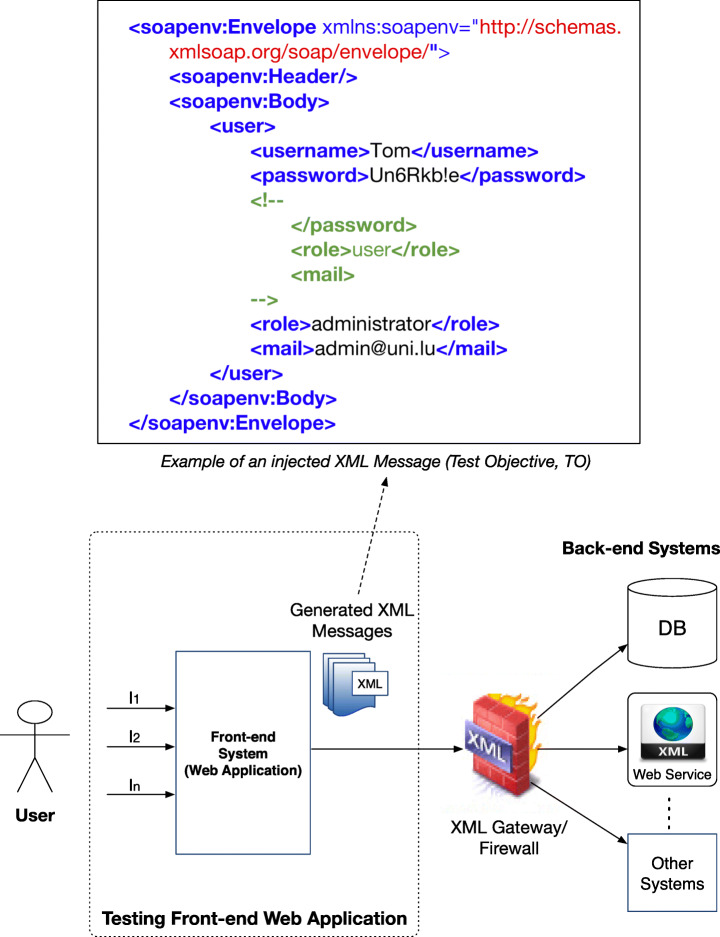
An example of XML-based Enterprise System

Enterprise systems are composed of several components (e.g., SOAP web services, web applications). Figure 1 depicts a typical three-tiered XML-based business application (Felderer et al. 2016). It consists of different components: front-end systems (typically web applications), an XML gateway/firewall, and the back-end web services or databases. In a typical scenario, the front-ends receive user inputs and generate XML messages, which are forwarded to the XML gateway/firewall. At this stage, malicious XML messages are filtered out while the benign ones are sent to the back-end web services (or databases). Attackers may exploit XML-based vulnerabilities at any tier, e.g., targeting the front-end web application or the XML gateway/firewall. However, the front-end web application is at most risk as an attacker can directly interact with it. If the front-end is vulnerable to *XMLi*, an attacker may produce and send malicious XML messages to the back-end web services.

*XMLi* attacks are the most common XML-based attacks that aim to manipulate or compromise the logic of a web application (Williams and Wichers 2013). They are carried out by injecting malicious strings into user inputs to produce harmful XML messages. This, in turn, can result in compromising the systems or subsequent components that receive and process the malicious XML messages. *XMLi* attacks can be used as a carrier for other types of attacks, such as SQL Injection, Cross-site Scripting, or Privilege Escalation (OWASP 2016). Their impact depends on the type of malicious content that the XML message carries, e.g., an attack can result in breaching confidential data.

To better understand *XMLi* attacks, let us consider an example of a web application for user registration (Jan et al. 2017a) that uses an XML database to store user registration data. Users can register via a web form by submitting three inputs: (i) *username*, (ii) *password*, and (iii) *mail*. The application assigns privileges to the user by generating a *role*, creates an XML SOAP message and sends it to the central service. Users are not allowed to modify the *role* element. We assume that the application directly concatenates the user inputs to the XML elements in the SOAP message. Figure 1 contains the resulting SOAP message for the following malicious inputs:

- *Username* = Tom
- *Password* = Un6Rkb!e</password><!--
- *E-mail* = --><role>administrator</role><mail>admin@email.com

As we can observe in the figure, the original (first) *role* element with the value of user is commented out and a new *role* element having the value of administrator is inserted in the message. In this way, the malicious user Tom has succeeded in escalating his privileges to the administrator level. Since this SOAP message is syntactically correct and is valid according to the associated schema, a validation procedure will not detect this vulnerability.

#### Testing Front-End Web-Applications for *XMLi*

Testing the input validation and sanitization procedures of front-ends is crucial to guarantee the security of the internal web services.

In our previous paper (Jan et al. 2017a), we proposed a black-box testing strategy targeting *XMLi* vulnerabilities. Such a strategy generates user inputs and inspects the corresponding XML messages produced by the given front-end web application, which corresponds to the actual software under test (SUT).

The basic idea is to test whether well-formed and yet malicious XML messages can be generated by front-ends given some specific user inputs, i.e., input strings of HTML forms. Given an XML message *X* known to be harmful to the internal web services, genetic algorithms (GAs) are used to search for input strings that —once validated and executed against the SUT— lead to the generation of *X*. If such input strings are found, it implies that input validation and sanitization are incomplete as they do not detect malicious inputs resulting in *XMLi* attacks.

*Coverage criteria*.: Since the goal is to find as many *XMLi* vulnerabilities as possible, multiple XML malicious messages have to be used as targets to cover various types of attacks. In the following, we refer to the set of malicious XML messages to target with GAs as Test Objectives (TOs), to be consistent with the terminology used in Jan et al. (2017a). TOs are defined based on four types of *XMLi* attacks (Jan et al. 2016), namely (i) *deforming*, (ii) *random closing tags*, (iii) *replicating*, and (iv) *replacing* attacks. Each of these attacks can have a different impact such as creating a malformed XML message to crash the system, nested attacks like SQL Injection or Privilege Escalation. We use an automated tool, namely *SOLMI*, to create a diverse set of TOs. *SOLMI* is specifically designed to generate malicious XML messages based on various types of *XMLi* attacks, and is very effective compared to other state-of-the-art tools (Jan et al. 2016, 2017a).
*Search algorithm*.: To enable the search for *XMLi*, Jan et al. (2017a) used a classical GA with *string encoding schema*. Given a set of TOs, the GA is executed multiple times, once for each TO (single-target strategy). Thus, the testing technique terminates when all TOs have been targeted by the GA.

A candidate solution (also called *chromosome* or *individual*) is a list of strings *I* = 〈*I*_1_,*I*_2_,…,*I*_*N*_〉 to insert in the target web-form, where *I*_*k*_ denotes the string for the k-th input of the SUT. The GA is initialized by generating a random pool of chromosomes, called *population*, which is evolved across various iterations (or *generations*). In each generation, the fittest chromosomes (*parents*) are selected and combined to form new chromosomes (*offsprings*) using *crossover* and *mutation*. More specifically, the *single-point crossover* creates new input strings by combining the input strings of the two selected parents; the *character mutation* randomly adds, deletes or changes some characters in the offsprings. The fitness of each chromosome *I* is measured by computing the *edit distance* (Alshraideh and Bottaci 2006) between the target TO and the message generated when executing *I* against the SUT. A zero edit distance value indicates that *Icovers* the target TO, i.e., the SUT generates the TO when executed using *I*. The GA terminates if either the target TO is covered or the maximum search time is reached.

A later variant of the aforementioned single-target strategy (Jan et al. 2017b) uses real-coded genetic algorithms rather than classical string-coded GAs. The overall idea is to consider characters forming input strings with the corresponding ASCII code. This allows the application of real-coded operators, such as the *single arithmetic crossover* and the *gaussian mutation*, that are known to work better than classical operators when dealing with numerical problems (Deb and Deb 2014). Finally, we also investigated the *real-coded edit distance* as a substitute of the *string edit distance* where the difference between characters is measured as the relative distance between their corresponding ASCII codes. The results of an empirical study with both open-source and industrial systems showed that the real-coded GA combined with real-coded edit distance is able to detect more *XMLi* vulnerabilities and in less time compared to other combinations of search algorithms and fitness function (Jan et al. 2017b).

*Limitations*. While using the real-coded search helped to improve the effectiveness and the efficiency in detecting *XMLi* attacks, it does not solve the *budget allocation* problem. Given a total search budget *B* to assess all possible TOs, each TO is assigned a local search budget equal to *B*_*TO*_ = *B*/|*TOs*|, where |*TOs*| is the total number of test objectives to cover. If one TO is covered and its local budget is not fully consumed, the search budget for the remaining uncovered TOs is dynamically recomputed, as the total remaining search budget divided by the yet uncovered TOs.

In such a scenario, the search budget is dynamically divided among the TOs. Therefore, the order by which the TOs are selected as targets may impact the search effectiveness, i.e., the number of TOs covered within the search budget *B*. Indeed, some TOs can be infeasible because the input validation routines of the SUT are able to prevent the generation of the malicious messages regardless of the input string inserted in the web forms. In addition, not all TOs require the same search budget to be covered: some TOs can be more expensive than others since, for example, they require more GA generations as the attack may involve multiple input parameters. If the less expensive TOs are selected first as targets, the saved search budget can be used to increase the budget assigned to the remaining TOs. Instead, infeasible TOs or TOs that cannot be covered within their local budget *B*_*TO*_ should not be targeted first as they represent an inefficient budget allocation. However, the feasibility or the time needed to cover each TO is a priori unknown. Therefore, managing the search budget allocation in an efficient way is very challenging.

In this paper, we devise the need for more advanced testing strategies that target all TOs at the same time, thus avoiding the inefficiency of single-target strategies.

### Multi-target Search-Based Techniques in White-Box Unit Testing

In the context of white-box unit testing, various strategies (Panichella et al. 2015, 2017; Fraser and Arcuri 2014) have been investigated in recent years aimed at overcoming the limitations of the single-target strategy. The key idea is considering all coverage targets (e.g., branches in white-box testing) as multiple independent objectives to optimize at the same time. Solving all objectives at once prevents the search from focusing on one single target (e.g., branch) that is infeasible or too difficult to cover within a given amount of time. Although recent research effort focused on unit testing only, the problem of covering multiple targets can be generalized for different types of testing, including *XMLi* vulnerability detection. Indeed, our goal is to generate multiple *XMLi* attacks, one for each target TO (malicious XML message).

In the following subsections, we briefly describe the most recent and effective multi-target testing techniques, as proposed in the context of white-box unit testing.

#### Many-Objective Sorting Algorithm

MOSA (Panichella et al. 2015, 2017) is a many-objective genetic algorithm that customizes NSGA-II (Deb et al. 2002), one of the most popular multi-objective genetic algorithms, for white-box testing. In MOSA, all coverage targets in white-box unit testing (e.g., branches) correspond to different objectives to optimize. Therefore, a chromosome is a test case and its fitness (optimality) is based on a vector of scalar values (objective scores) capturing the distances from all uncovered targets (e.g., uncovered branches). To handle the potentially large number of targets (objectives) in a program, MOSA uses two *preference criteria* to select and evolve (in the next iterations) a subset of test cases in the Pareto front. This subset should contain the test cases with minimum distance for each uncovered target and, when multiple test cases show the same distance, shorter test cases should be selected. The distance for each test *τ* is measured according to the type of coverage targets (Panichella et al. 2017). For branch coverage, it is the sum of the normalized branch distance (McMinn 2004a) of *τ* for branch *b*_*i*_ and the corresponding approach level (McMinn 2004a).

To further speed-up the search, the set of objectives to optimize in MOSA at each generation is kept dynamic and corresponds to the yet uncovered targets. Test cases satisfying some of the branches are stored within a second population, called *archive*. The *archive* is updated as soon as a new test *τ* is generated depending on whether (i) it satisfies previously uncovered targets or (ii) it is shorter than another test *τ*^∗^ in the archive, which covers the same targets (i.e., *τ* and *τ*^∗^ are equivalent regarding coverage but the former contains fewer statements than the latter).

With the exception of these three components (i.e., preference criteria, dynamic selection of the targets, and archiving strategy), MOSA shares the same main loop with NSGA-II (or any other GA). Indeed, the initial population is iteratively evolved using *mutation* and *crossover* while the selection is based on the preference criteria. At the end of the search, the final test suite corresponds to the updated *archive* from the last generation.

In the context of *XMLi*, we notice that the original MOSA algorithm cannot be directly applied for two main reasons. First, in traditional white-box unit testing, it is very frequent that two or more test cases with different lengths are equivalent in terms of objective scores (i.e., same coverage). Therefore, prioritizing shorter tests at the same level of coverage may help in generating better (more concise) tests. In the context of *XMLi*, a target TO can be covered by only one single solution (input strings) and other equivalent shorter strings cannot exist. Second, the crossover operator is detrimental if it recombines two different chromosomes that are optimizing two different TOs. For example, let us assume that MOSA selects as parents the two chromosomes *I*_1_ = 〈OR 1〉 and *I*_2_ = 〈--><role>admn</role>〉. The former has an edit distance of *d*(*I*_1_,*TO*_1_) = 2 for the test objective *TO*_1_ = “<test>data OR 1 = 1</test>”; the latter is the closest chromosome covering the test objective *TO*_2_ =“--><role>admin</role><mail>admin@email.com” with a distance *d*(*I*_2_,*TO*_2_) = 1. Applying the single point crossover to recombine *I*_1_ and *I*_2_ will result in offsprings having worse edit distances for both *TO*_1_ and *TO*_2_. In other words, the crossover is damaging the original input strings in terms of satisfying uncovered TOs.

To make MOSA applicable in the context of *XMLi*, we developed a variant, which we call vMOSA. Such a variant shares the main loop with the original MOSA but it differs on the following two points: (i) the preference criterion does not include the length of the chromosomes as a secondary objective; (ii) for the reasons explained above, offsprings are generated by only using the mutation operator (i.e., the crossover operator is not used).

Please notice that an extension of MOSA, called DynaMOSA, has been recently proposed in the literature (Panichella et al. 2017). It uses control flow analysis to reduce the number of targets to optimize in each generation. Although being more effective than MOSA in white-box unit testing, DynaMOSA cannot be applied for *XMLi* testing as no structural dependencies exist among the different TOs to cover.

#### Many Independent Objective Algorithm

The Many Independent Objective (MIO) algorithm (Arcuri 2017) is an evolutionary algorithm designed to improve the scalability of test suite generation for non-trivial programs with a very large number of testing targets (e.g., in the order of thousands/millions). It is tailored around the following three main assumptions in white-box testing: (i) testing targets (e.g., lines and branches) can be sought independently, as test suite coverage can be increased by adding a new test case; (ii) testing targets can be either strongly related (e.g., nested branches) or completely independent (e.g., when covering different parts of the SUT); (iii) some testing targets can be *infeasible* to cover.

Based on the above assumptions, at a high level, the MIO algorithm works as follows: it keeps one population of tests for *each* testing target (e.g., branches). Individuals within a population are compared and ranked based on their fitness value computed *exclusively* for that testing target. At the beginning of the search, all populations are empty and are iteratively filled with generated tests. At each step, with a given certain probability, MIO either samples new tests at random or samples (and then mutates) one test from one of the populations related to uncovered targets. A sampled test is added to *all* the populations for uncovered targets and is thus evaluated and ranked independently in each population. Once the size of a population increases over a certain threshold (e.g., 10 test cases), the worst test (based on its fitness for that population) is removed. Whenever a target is covered, its population size is shrunk to one, and no more sampling is done from that population. At the end of the search, a test suite is created based on the best tests in each population.

*Feedback-directed sampling*.: For each population, there is a counter, initialized to zero. Every time an individual is sampled from a population *X*, its counter is increased by one. Every time a new, better test is successfully added to *X*, the counter for that population is reset to zero. When sampling a test from one of the populations, the population with the lowest counter is chosen. This helps focus the sampling on populations (one per testing target) for which there has been a recent improvement in the achieved fitness value. This is particularly effective to prevent spending significant search time on infeasible targets (Arcuri 2017).
*Parameter-control*.: To dynamically balance the tradeoff between *exploration* and *exploitation* of the search landscape, MIO changes its parameters during the search (similarly to Simulated Annealing).

### Cooperative Co-Evolutionary Algorithms

Co-evolutionary algorithms extend more classical genetic algorithms by evolving multiple populations (Potter and De Jong 1994) (often referred to as *islands* or *species*) rather than one single population of solutions. The overall idea consists of solving complex problems by using the principle of *divide and conquer* (Goh and Tan 2009): a large problem is divided into many sub-problems; an *island* (or sub-population) is initialized and evolved for each sub-problem separately; finally, the solution to the original problem is obtained by assembling the best solutions from each island (specie).

Each island is evolved separately using standard genetic algorithms, i.e., selection, crossover and mutation are used to recombine solutions (parents) within the same islands to create new solutions (offsprings). Each solution is assigned a local fitness score that measures its ability to solve the sub-problem (island) it belongs to.

While islands are evolved separately, they interact with each other through periodical migration (Bali and Chandra 2015; Keerativuttitumrong et al. 2002; Wassermann and Su 2007), which is a genetic operator specific to cooperative co-evolutionary algorithms. It consists of copying and injecting the strongest solution from one of the islands to the other ones with the goal of increasing genetic diversity and supporting islands with poor performance (Bali and Chandra 2015) (e.g., no improvements in local fitness scores). During migration, the island with the largest fitness improvements in local fitness score is selected and the strongest solution from that island is copied into the other islands. If two or more islands are equally eligible for selection (i.e., multiple islands have local fitness improvements), the winner can be selected randomly among them.

## A New Approach

Test generation for detecting *XMLi* vulnerabilities features important differences with respect to white-box unit testing. First, coverage targets in white-box testing (e.g., branches) are organized into a priority hierarchy according to their positions in the control flow graph (CFG) (Panichella et al. 2017). For example, in a program with two nested if conditions, the branches of the inner if condition can be covered if and only if the outer condition is already satisfied. Instead, in security testing, the target TOs are completely independent of each other and there is no structural relationship among them, i.e., covering one TO does not depend on whether any other TO has been covered previously. Another important difference relates to the *collateral coverage* phenomenon. In white-box testing, some targets (e.g., branches) can be accidentally covered when optimizing other coverage targets (McMinn 2004b; Arcuri et al. 2012). In the context of *XMLi* attack generation, collateral coverage never happens given the fact that no relationship exists among different TOs.

**Fig. 2:**
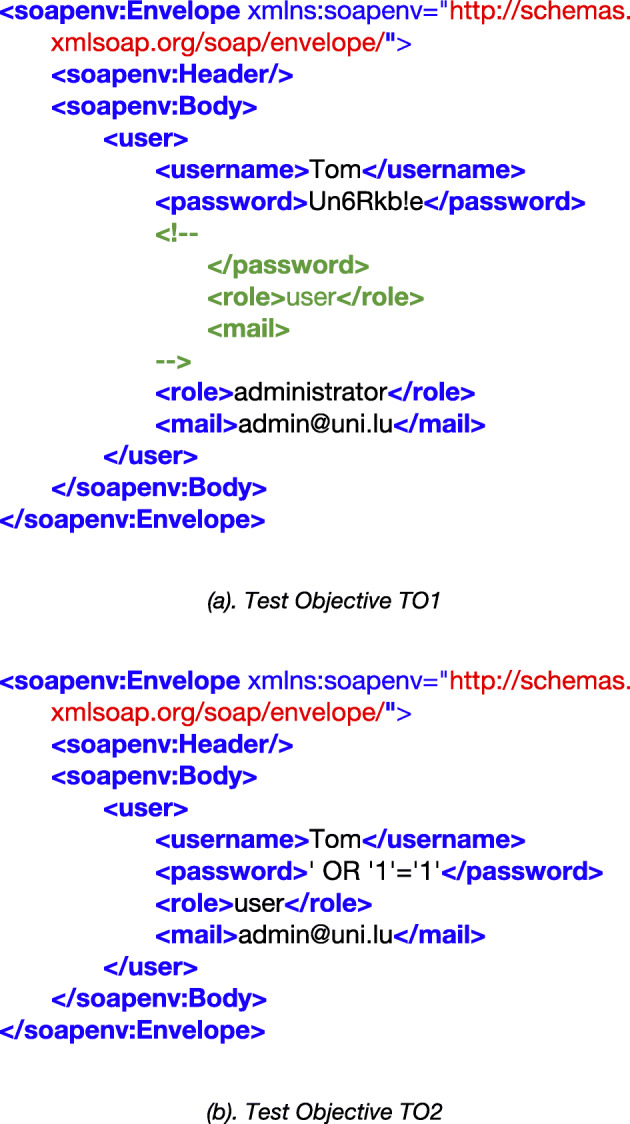
Example of Test Objectives (TOs)

To better explain why the TOs are independent of each other, let us consider as an example the two TOs, TO1 and TO2, shown in Fig. 2. The two TOs correspond to two different types of XML Injection attack as described in Jan et al. (2016). The SUT can generate TO1 only with the following inputs:

- *Username* = Tom
- *Password* = Un6Rkb!e</password><!--
- *E-mail* = --><role>administrator</role><mail>admin@uni.lu

Therefore, to cover TO1, the search algorithm must find these unique inputs. As shown in the figure, the malicious content in TO2 is different from TO1. To cover this TO, the following inputs are needed:

*Username* = Tom

*Password* = ' OR'1'='1'

*E-mail* = admin@uni.lu

Although the Username input is similar for these two TOs, the other two inputs (Password and Email) are entirely different. Finding the three inputs for TO1 does not depend on the inputs or coverage of TO2 and vice versa. Also, these TOs can only be covered with their corresponding unique inputs as mentioned above. Further, since each TO requires the unique combination of the three inputs, it is not possible to accidentally cover a TO with the inputs of another TO during the search.

Starting from these observations, we propose a novel many-objective, co-evolutionary algorithm that is customized for *XMLi*. To further speed-up the search process, we also describe an alternative fitness function with a lower computational complexity compared to the commonly-used string edit distance (Jan et al. 2017a). While we demonstrate that the proposed methodology is effective and efficient for *XMLi*, we believe that the novel algorithm and fitness function can be adapted or reused to other types of injections attacks.

The details of the novel search algorithm and fitness function are described in the next subsections.

### Cooperative Co-evolutionary Algorithm for *XMLi*


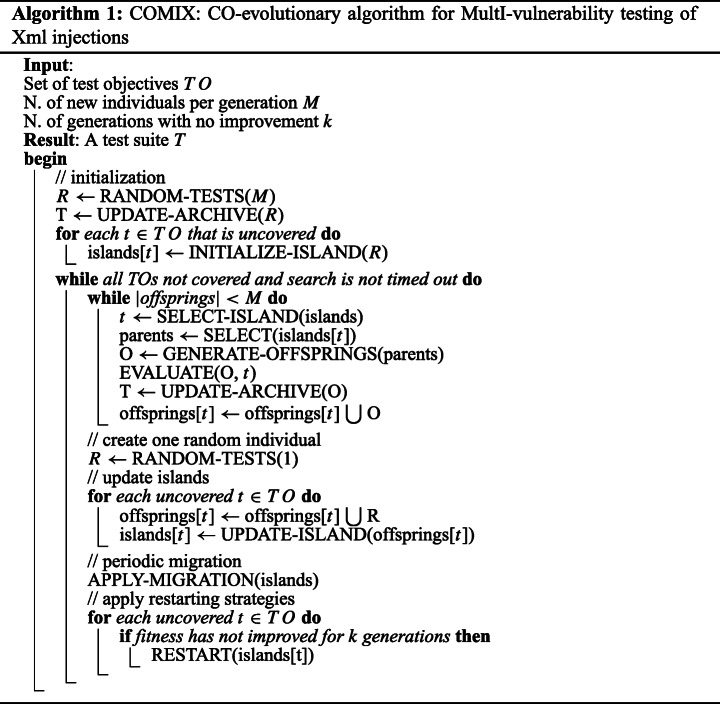


In this paper, we introduce a novel many-objective, cooperative, and co-evolutionary algorithm tailored for *XMLi*, hereinafter referred to as COMIX (*CO-evolutionary algorithm for MultI-vulnerability testing of Xml injections*).

In our context, the overall problem can be formulated as *generating XMLi attacks that match/cover all target TOs*. This problem can be divided into sub-problems: *generating one test case (attack) for each target TO*. Therefore, in a co-evolutionary environment, each TO corresponds to an island to evolve. Once a given sub-problem is solved (i.e., an attack has been generated for its corresponding TO), its test case is stored in the final test suite. Therefore, at the end of the search, the test suite will contain all successfully attacks generated across search iterations.

The pseudo-code of COMIX is detailed in Algorithm 1. COMIX initializes the search by randomly generating a set of test cases *R* (line 3), which is used to initialize the islands (loop in lines 5-6). For each target *t*, an island *islands[t]* is created using the routine INITIALIZE-ISLAND (line 6). Such a routine (i) sorts *R* in ascending order of fitness value (distance) for *t*; and (ii) it copies the top *μ* ⊂ *R* tests in the corresponding *islands[t]*. After this initialization process, the islands are evolved independently through subsequent iterations within the loop in lines 7-26.

In each iteration, islands are evolved separately using three traditional genetic operators: *selection*, *crossover*, and *mutation*. Given an uncovered target *t* ∈ *TO*, two parents are selected from the corresponding island *islands[t]* using the *binary tournament selection*. Then, the two parents are recombined using *crossover* and *mutation* (routine GENERATE-OFFSPRINGS in line 11) forming two offsprings. These offsprings are evaluated only against the test objective *t* ∈ *TO* and are inserted into an offspring island *offsprings[t]*. At the end of each iteration, the total number of new individuals (test cases) generated across the islands is kept constant (condition in line 8): *M* − 1 new tests are created using the routines GENERATE-OFFSPRINGS; the last solution is randomly generated (line 16) to reach the set population size M and preserve diversity.

*Islands selection*. There are multiple islands from which we could select and recombine solutions in each generation.

In COMIX, we use a heuristic similar to the feedback-directed sampling used in MIO (Arcuri 2017) (the routine SELECT-ISLAND in line 9). More specifically, islands with recent improvements in their fitness function have a higher likelihood of being selected for evolution. For each island *islands[t]*, COMIX uses a counter to keep track of the number of times an island was selected in past generations and the new generated tests did not lead to any improvements for the corresponding test objective *t*. Every time the fitness function for *t* is improved (decreases) the corresponding counter is reset to zero. Such a counter is used to assign a selection probability to each island associated with an uncovered TO. Let *C*(*t*_*i*_) be the value of the counter for the island *island[t*_*i*_*]*, its probability of being selected for evolution is computed as:

1 $$ p(t_{i}) = \frac{1}{C(t_{i})+ 1} \times \frac{1}{\sum\limits_{t_{j} \in TO} \frac{1}{C(t_{j}) + 1}} $$

In other words, the larger the value of the counter *C*(*t*_*i*_), the lower the probability for the *island[t*_*i*_*]* to be selected. This heuristic helps to focus the search towards promising islands and to penalize those with no improvements in recent generations.

*Updating the islands*.: At the end of each iteration, the island of each uncovered target *t* is updated with the new individuals stored in the corresponding offspring island *offsprings[t]* defined for the same target *t* (lines 18-20). In particular, the routine UPDATE-ISLANDS sorts parents and offsprings (that compete with each other) according to the fitness function for the given island and the top *μ* tests survive for the next evolutionary iteration. In addition, the random test generated in line 16 is copied to each island *offsprings[t]* and competes with offsprings and parents when forming the island for the next iteration.
*Migration policy*.: Although the islands are evolved independently, migration strategies are applied in co-evolutionary algorithms in order to migrate (copy) the strongest individuals in one source island and replace the weaker one in a target island (Keerativuttitumrong et al. 2002; Tan et al. 2006). The motivation is that one good solution in an island might turn out to be good in another island as well.

In our context, such a strategy might be effective since, though TOs are independent, some of them might share some commonalities, such as common substrings needed to evolve for the inputs. For example, when the SUT uses input validation techniques, it produces error messages when the user-supplied data does not conform to a specific rule set. In such a scenario, randomly generated input data (test cases) lead to error messages during the initial stages of the search. When one island produces the first test case that passes the input validation, the SUT produces an XML message that is used to compute the fitness function (distance). This passing test case is useful not only for the island it belongs to but also for all other islands to evolve.

On the one hand, a migration policy would help spread such good substrings among the different islands. On the other hand, a too high migration rate could be detrimental, as it would also share genetic material that is only good for a specific island. Based on our preliminary results, we found that migrating one single test case per search iteration leads to a higher percentage of covered TOs. An analysis on the performance of COMIX with different *migration rates* is reported in Section 5.4.

In Algorithm 1, the migration is performed in line 22 using the routine APPLY-MIGRATION. Such a routine randomly selects one uncovered test objective *t*, copies the best test case from the corresponding island *islands[t]* into all the other islands *islands[t*^′^*]* (with *t*^′^≠*t*) and, evaluates it against the corresponding TOs. APPLY-MIGRATION selects the test case to migrate exclusively from islands that have improved in recent iterations. This is meant to avoid repeating the same migrations over iterations and prioritizing the migration of new, good solutions in recently improved islands.

*Archiving*.: Following the search strategy implemented in MIO and MOSA (Panichella et al. 2015, 2017), COMIX focuses the search only on the uncovered TOs (see lines 9, 18, and 24). Test cases satisfying previously uncovered TOs are stored into an *archive* (Panichella et al. 2015, 2017), which is an external data structure representing the final test suite. The archive is updated by the routine UPDATE-ARCHIVE whenever new test cases are generated (lines 4 and 13).
*Re-starting strategies*.: Restarting the search is a common practice in evolutionary algorithms to reduce the probability of converging toward local optima (Jansen 2002). For this purpose, COMIX restarts the islands for which stagnation is detected (line 25 of Algorithm 1). Stagnation is detected separately for each island when the fitness function (distance to the corresponding TO) of the best test case within the island has not improved in the latest *k* subsequent iterations. Islands satisfying the condition in line 25 are restarted, i.e., its *μ* individuals are deleted and replaced with randomly generated tests (routine RESTART in line 26).
*Differences with other multi-target strategies*.: MIO, vMOSA and COMIX target all TOs at once. However, there is a substantial difference in how they evaluate the chromosomes. In vMOSA, all TOs are objectives to optimize in a many-objective scenario; therefore, each individual is evaluated against all the uncovered TOs (i.e., the edit distance is computed for each uncovered TO). Even if MIO uses different populations (one for each TO), it still performs multiple edit distance computations, one for each uncovered TO. Instead, in COMIX, the TOs are completely independent and, thus, each individual is evaluated only against the single TO optimized by the island it belongs to. In other words, COMIX performs one single edit distance computation per individual.

Another important difference is that COMIX uses the crossover operator while MIO and vMOSA do not. However, it is worth noting that in COMIX the crossover is applied within each *island* and therefore it is used to recombine chromosomes optimizing the same target TO. Instead, MIO does not use the crossover by design (Arcuri 2017) while in vMOSA we had to disable the crossover because it is detrimental when recombining chromosomes optimizing different TOs (see Section 2.2.1).

### Linear Complexity Fitness Function

The original fitness function used by Jan et al. (2017a) is the string edit distance (or Levenshtein distance), which is the standard string fitness function used in search-based testing (Alshraideh and Bottaci 2006). Given two strings *A* and *B*, the *edit distanced*(*A*,*B*) is equal to the minimum number of characters to insert, delete and change in *A* to obtain *B*. In our previous paper (Jan et al. 2017b), we improved the edit distance with a real-coded variant where, whenever a character c1 is substituted with a character c2, the overall distance is increased by the difference of the ASCII codes of c1 and c2. In Sections 4 and 5, we explain why and show how such a modification provides additional guidance to the search.

A potential limitation of the edit distance is its high computational cost, which is $\mathcal {O}(n \times m)$, with *n* and *m* being the lengths of the strings being compared. When using multi-target strategies for testing *XMLi* vulnerabilities, evaluating each chromosome can be very expensive when using MIO or vMOSA since it requires to compute the edit distance against each yet uncovered TO. In this paper, we consider a less expensive fitness function; given two strings *A* (with length *n*) and *B* (with length *m*), their distance is defined as:

2 $$ d(A, B) = |n - m| + \sum\limits_{i = 1}^{\min\{m,n\}} \frac{|a_{i} - b_{i}|}{|a_{i} - b_{i}| + 1} $$

where *a*_*i*_ and *b*_*i*_ denote the ASCII codes for the characters in position *i* of *A* and *B*, respectively. With its first term, (1) strongly penalizes differences in lengths among strings. The second term penalizes differences in characters in the shortest string by accounting for character differences in ASCII code. Such a difference is normalized to be always inferior to missing characters due to different lengths. The usage of the character differences in ASCII code has been proposed in previous studies (Jan et al. 2017b; Alshraideh and Bottaci 2006) and provide better guidance than search based on the classical edit distance.

In the following, we refer to the distance in (2) as *linear distance* since its computational complexity is $\mathcal {O}(\min \{n, m\})$. In our empirical evaluation, we compare the linear distance with the *real-coded edit distance* (Jan et al. 2017b), which has been proven to be more effective (i.e., provide better guidance) than the classical edit distance. Though the linear distance is definitely less expensive to compute than the real-coded edit distance, it provides less guidance to the search and is more exposed to getting stuck in local optima. This is why an extensive empirical comparison is required.

## Empirical Study

This section describes our empirical evaluation, whose goal is to assess our proposed search-based approach and compare it with state-of-the-art testing strategies for XML Injection.

### Study Context

We carried out our evaluation on different versions of four web applications, namely SBANK, SecureSBANK (SSBANK), XMLMAO and M.

The first two subjects are XML-based web applications interacting with a real-world bank card processing system of a credit card processing company. They are simplified versions of the actual front-end web applications from one of our industrial collaborators (a credit card processing company^1^).

Both SBANK and SSBANK have three versions with a different number of user inputs, i.e., SBANK1 (SSBANK1), SBANK2 (SSBANK2) and SBANK3 (SSBANK3). These different versions of the same applications are used to analyze to what extent the number of input parameters affects the ability of solvers and fitness functions to detect XMLi vulnerabilities. Each application version receives user inputs, produces XML messages, and sends them to the back-end web services. All versions of SBANK are vulnerable to XML Injections as they do not apply any input validation or sanitization routine on user inputs. On the other hand, SSBANK applications contain validation and sanitization procedures for one of its user inputs (i.e., *IssuerBankCode*) that are applied before generating the XML messages.

The third subject of our study is a vulnerable-by-design, open-source web application, namely XMLMao (Magical Code Injection Rainbow (MCIR) 2016). It is a module of the *Magical Code Injection Rainbow (MCIR)* - a framework for building a configurable vulnerability test-bed and is available on GitHub.^2^

The fourth subject M is an industrial web application with millions of registered users and hundreds of thousands of visits per day. The application itself is hundreds of thousands of lines long, communicating with several databases and more than 50 corporate web services (both SOAP and REST). Out of hundreds of different HTML pages served by M, in this paper we focus on one page having a form with two string inputs. As the experiments on this system had to be run on a dedicated machine (e.g., it could not be run on a research cluster of computers) due to confidentiality constraints, we could not use all of its web pages and forms. We chose one example manually, by searching for non-trivial cases (e.g., web pages with at least two string input parameters that are not enumerations), albeit not too difficult to analyze, i.e., given the right inputs, it should interact with at least one SOAP web service. Due to non-disclosure agreements and security concerns, no additional details can be provided on M.

The selected systems have varying size and complexity, are written using different programming languages and technologies (i.e., Java and PHP) and interact with a variety of back-end web services. In addition, these web applications differ in the number of user inputs as well as their processing routines: SBANK and XMLMAO have no input validation or sanitization, while SSBANK and M use various routines to validate and sanitize user inputs. Moreover, all these web applications have already been used in the literature (Jan et al. 2017a) to assess the effectiveness of search-based testing techniques for *XMLi* detection.

*Test Objectives Generation*.: In our testing context, a Test Objective (TO) is an XML message with malicious content that may result into an XMLi attack on the back-end web services.

For each subject application, we created 50 Test Objectives (TOs) based on different types of *XMLi* attacks (Jan et al. 2016). These TOs are created using SOLMI (Jan et al. 2016), an automated tool designed for generating successful *XMLi* attacks. We selected this tool as it outperforms state-of-the-art attack generation tools (Jan et al. 2017a). Moreover, it creates malicious XML messages (test objectives) covering the four most common and critical types of *XMLi* attacks that, if generated by the front-ends, could compromise the back-end services.

### Research Questions

In this paper, we investigate the following three research questions:

**RQ1:** *What is the best search-based algorithm for generating XMLi attacks?* This research question aims at finding the most effective and efficient algorithm for detecting *XMLi* vulnerabilities. In particular, we compare the performance of the proposed COMIX algorithm with vMOSA, MIO, and the single-target strategy, while using two different distance functions.
**RQ2:** *Is the execution time to achieve maximum coverage for a given set of TOs acceptable in practice?* We investigate the performance of COMIX, which is the best approach according to the results from RQ1, from the perspective of security analysts who want to uncover as many *XMLi* vulnerabilities as possible within practical execution time.
**RQ3:** *What is the impact of using the linear distance on the fitness calculation time?* This research question investigates the impact of the alternative fitness function proposed in this paper (linear distance) on the time needed to evaluate candidate solutions. This is intended to better explain the results in RQ1. Therefore, we compare the amount of time spent on fitness calculations by COMIX for the two fitness functions: edit distance and linear distance.

To answer the research questions above, we use the following two performance metrics: *Coverage* and the *Area Under the Curve (AUC)*.

*Coverage (C)* is the ratio |*Covered*|/|*Feasible*|, where *Covered* denotes the TOs covered by a given algorithm, while *Feasible* is the set of feasible TOs. To determine the feasible TOs, we carefully inspected the source code of the front-ends, their input validation and sanitization routines, and we analyzed each TO generated by SOLMI. Notice that all evaluated testing strategies are black-box and therefore do not require access the source code. We performed this analysis only for the purpose of computing the coverage scores. We found that all 50 TOs generated by SOLMI for the SBANK versions and for XMLMAO are feasible. In contrast, the number of feasible TOs for all SSBANK versions is 34 (out of 50), whereas for M it is only 2.

While coverage is typically used to assess effectiveness at the completion of the search, analyzing coverage over time provides more fine-grained information about the efficiency of each algorithm. The simplest methodology to perform such an analysis consists of plotting coverage over running time, for each iteration/generation of the compared algorithms (*coverage graph*). To better quantify the differences among algorithms, we use *AUC* of the coverage graphs, computed according to the *trapezoidal* rule (Davis and Rabinowitz 2007). The AUC is a scalar value in the range [0, 1]; higher AUC values indicate that an algorithm achieves higher coverage in less execution time. Since the search time used in our experiment is to some extent arbitrary and that, in practice, people may have less time than required to achieve maximum coverage, AUC provides a useful additional indicator about what search strategy is to be selected.

### Experimental Procedure

We carried out a number of experiments on each version of the web applications with the four algorithms considered in this paper. For each experiment, and for each algorithm run, we recorded the time needed to cover each TO (if covered), the total execution time, the time spent on the fitness calculations, and the time required for executing tests. All execution times are recorded in minutes.

To account for the randomized nature of the optimization algorithms and to conduct a reliable statistical analysis, we ran each algorithm 30 times on each version of the subject applications. There are total seven versions of our open source subjects, i.e., three for SBANK, three for SSBANK and one for XMLMao. We allocated 30 minutes to each experiment resulting in an execution time of 420 hours^3^ for all experiments. Since all these experiments had to be run twice, i.e., for 5 and 50 TOs, it would normally result in 840 hours of total execution time. To reduce it to a manageable time, we used a cluster of computers. A separate virtual machine (node) was dedicated for the experiments involving each application version. Hence a total of 14 nodes were used which reduced the total execution time from 840 hours to 60 hours. In contrast, the experiments on the industrial case study M had to be run on a dedicated physical machine, and were repeated only 10 times.

For answering **RQ1**, we first analyzed the coverage and the AUC values obtained by each algorithm. Next, we applied the Friedman’s test (García et al. 2008) to verify whether the differences among the algorithms are statistically significant. The Friedman’s test is a non-parametric test for multiple-problem analysis and it is the most suitable statistical test for comparing different randomized algorithms when considering multiple benchmarks (García et al. 2008), i.e., the software systems in our case. This test has been used in various CEC competitions (e.g., Chen et al. (2014)) and in the latest SBST competition (Panichella and Molina 2017) to compare evolutionary algorithms and testing tools. For the level of significance, we used *α*= 0.05. While the Friedman’s test indicates whether a group of algorithms are significantly different, a statistical test for multiple pairwise comparisons is needed to understand which pair of algorithms are significantly different in terms of AUC values. To this aim, we used the pairwise Wilcoxon test with a significance level of *α*= 0.05. Because of the multiple comparisons, the *p*-values of the Wilcoxon test were further adjusted using the Holm-Bonferroni procedure (Holm 1979) for correcting the significance level.

To answer **RQ2**, we analyzed the execution time required to achieve maximum coverage with COMIX and assessed the practical usability of our approach in a realistic context. To this aim, we collected the time at which each TO is covered in a given run; then, we computed the elapsed time between the beginning of the search and the time in which we detect the last covered TO. Notice that, in practice, security analysts may stop the search before consuming the entire search budget if no further improvement is observed in the distance values for all uncovered TOs.

To answer **RQ3**, we investigated the execution time of the fitness function computations. For each subject, we compared the execution time spent on the fitness calculation when using the two distance functions, i.e., the edit and the linear distances. This analysis helped us understand the magnitude of the benefits obtained from using the linear distance over the traditional edit distance.

### Parameter Settings

We follow the recommendations in the related literature for setting the parameter values of the search algorithms, as detailed below:

- *Population size:* for the single target algorithm and for vMOSA we use a population size of 50 as recommended by recent studies in search-based software testing (Arcuri and Fraser 2013; Panichella et al. 2015, 2017). For MIO, the size of each population was set to 10 individuals (Arcuri 2017). Finally, in COMIX the size of each island is dynamically computed in each generation as:
  3 $$ \lambda = round(\#\text{Total Size} / \# \text{Uncovered TOs})+ 1 $$
  where *#* Total Size denotes the total number of test cases generated in each iteration of COMIX. For a fair comparison with vMOSA, we set *#* Total Size to 50 test cases.
- *Mutation:* It has been established in the literature (Briand et al. 2006; Schaffer et al. 1989; Smith and Fogarty 1996; Haupt and Haupt 2004) that a mutation rate based on population size and chromosome length achieves better performance. We confirmed this in our context with some preliminary experiments comparing this strategy with other mutation rates recommended in De Jong (1975) and Grefenstette (1986). Therefore, we use $p_{m}=(1.75)/(\lambda \sqrt {l})$ as mutation rate, where *l* is the length of the chromosome and *λ* is the population size.
- *Crossover:* We use the same crossover rate of 0.8 for the single-target approach as used in its original implementation (Jan et al. 2017a). As discussed above, for the many-objective algorithms considered in this paper, we do not apply crossover.
- *Search Timeout:* For each experiment on the open-source systems, we allocate a search budget of 30 minutes. For the industrial system M, we use a budget of 180 minutes, as each test execution takes much longer. The search also stops when all feasible TOs are covered.

Regarding the other configuration parameters of the experiments, we followed the settings that were empirically found superior in the original implementation of the single-target approach (Jan et al. 2017a). In particular, we used an initial population consisting of strings with variable lengths. Further, for generating input strings, we used a reduced alphabet set consisting of only the characters found in the TOs instead of the complete alphabet of all possible characters.

## Results

This section describes the results of our empirical evaluation to answer the research questions defined in Section 4.2.

### RQ1: What is the Best Search-Based Algorithm for Generating XMLi Attacks?

Tables 1 and 2 show the coverage results of each algorithm when using the edit and the linear distances, respectively. The *AUC* results are shown in Tables 3 and 4.

**Table 1:** Coverage achieved when using the edit distance (bold numbers indicate best results across techniques)

System	# Inputs	# TOs	COMIX		MIO		vMOSA		Single	
			Mean	Sd	Mean	S.d	Mean	S.d	Mean	S.d
SBANK	1	5	1.0000	−	1.0000	−	1.0000	−	1.0000	−
	2	5	**1.0000**	−	0.8800	0.1627	0.9933	0.0365	0.9600	0.0968
	3	5	**0.9867**	0.0507	0.8467	0.2446	0.7533	0.2270	0.8867	0.1252
SSBANK	1	5	1.0000	−	1.0000	−	1.0000	−	1.0000	−
	2	5	**1.0000**	−	0.9800	0.0610	1.0000	−	0.1000	0.1365
	3	5	**1.0000**	−	0.8000	0.4068	0.9400	0.2298	0.0333	0.0758
XMLMAO	1	5	1.0000	−	1.0000	−	1.0000	−	0.9933	0.0365
SBANK	1	50	**1.0000**	−	0.0140	0.0196	0.0413	0.0389	0.2860	0.1937
	2	50	**0.4553**	0.0918	0.0006	0.0037	−	0.0000	0.0467	0.0579
	3	50	**0.2120**	0.0582	0.0000	−	0.0000	−	0.0000	−
SSBANK	1	50	**0.9971**	0.0118	0.0108	0.0238	0.0373	0.0540	0.1069	0.0554
	2	50	**0.8160**	0.0482	0.0284	0.0350	0.0275	0.0362	0.0000	−
	3	50	**0.4108**	0.1011	0.0000	−	0.0000	−	0.0000	−
XMLMAO	1	50	**1.0000**	−	0.6153	0.0985	0.3813	0.0908	0.3247	0.0374
M	2	50	**0.7777**	0.2635	0.6500	0.3374	0.6500	0.2415	0.0000	−

**Table 2:** Coverage achieved when using the linear distance (bold numbers indicate best results across techniques)

System	# Inputs	# TOs	COMIX		MIO		vMOSA		Single	
			Mean	Sd	Mean	S.d	Mean	S.d	Mean	S.d
SBANK	1	5	**1.0000**	−	0.9800	0.0610	**1.0000**	−	0.8467	0.1871
	2	5	**1.0000**	−	0.9333	0.1213	0.9733	0.0691	0.1200	0.1243
	3	5	**1.0000**	−	0.6400	0.1610	0.8600	0.1192	0.0133	0.0507
SSBANK	1	5	1.0000	−	1.0000	−	1.0000	−	0.8200	0.1846
	2	5	**1.0000**	−	0.5067	0.3226	0.9933	0.0365	0.0067	0.0365
	3	5	**0.8813**	0.2583	0.0267	0.1142	0.8800	0.0997	0.0000	−
XMLMAO	1	5	**1.0000**	0.0000	0.9667	0.0758	**1.0000**	−	0.8133	0.1814
SBANK	1	50	**1.0000**	−	0.9807	0.0388	**1.0000**	−	0.6860	0.0536
	2	50	0.9320	0.0469	0.9693	0.0355	**0.9667**	0.0384	0.1293	0.0489
	3	50	**0.9716**	0.0434	0.5133	0.1342	0.9407	0.0350	0.0307	0.0221
SSBANK	1	50	**0.9117**	−	0.9961	0.0128	0.8637	0.1762	0.6824	0.0879
	2	50	**0.4067**	0.2628	0.3039	0.1272	0.2167	0.2817	0.0020	0.0075
	3	50	**0.3510**	0.4025	0.1265	0.1983	0.2686	0.3977	0.0000	−
XMLMAO	1	50	**1.0000**	−	0.8247	0.1217	0.9967	0.0130	0.3967	0.0847
M	2	50	0.2777	0.44095	**0.8500**	0.2415	0.4500	0.1581	0.0000	−

**Table 3:** AUC achieved when using the edit distance (bold numbers indicate best results across techniques)

System	# Inputs	# TOs	COMIX		MIO		vMOSA		Single	
			Mean	S.d	Mean	S.d	Mean	S.d	Mean	S.d
SBANK	1	5	**0.9759**	0.0039	0.8122	0.0283	0.8817	0.0271	0.9689	0.0037
	2	5	**0.9131**	0.0126	0.4945	0.1008	0.6236	0.0589	0.8878	0.1099
	3	5	**0.8665**	0.0465	0.3485	0.1258	0.2824	0.1053	0.8209	0.1165
SSBANK	1	5	0.9781	0.0068	0.8369	0.0286	0.9062	0.0166	**0.9790**	0.0060
	2	5	**0.9419**	0.0190	0.8131	0.1069	0.8421	0.0342	0.1340	0.1755
	3	5	**0.9117**	0.0242	0.5744	0.2934	0.6165	0.1692	0.0245	0.0646
XMLMAO	1	5	**0.9696**	0.0057	0.9044	0.0161	0.9165	0.0209	0.9609	0.0367
SBANK	1	50	**0.5637**	0.0590	0.0010	0.0014	0.0142	0.0166	0.2829	0.1919
	2	50	**0.1412**	0.0394	0.0001	0.0003	0.0000	0.0000	0.0459	0.0570
	3	50	**0.0540**	0.0182	0.0000	−	0.0000	−	0.0000	−
SSBANK	1	50	**0.6007**	0.0582	0.0018	0.0043	0.0094	0.0150	0.1054	0.0547
	2	50	**0.3497**	0.0399	0.0071	0.0107	0.0061	0.0103	0.0000	−
	3	50	**0.1185**	0.0348	0.0000	−	0.0000	−	0.0000	−
XMLMAO	1	50	**0.7983**	0.0351	0.3788	0.0496	0.2903	0.0516	0.3250	0.0407
M	2	50	**0.3450**	0.1646	0.2292	0.1288	0.3013	0.1018	0.0000	−

**Table 4:** AUC achieved when using the linear distance (bold numbers indicate best results across techniques)

System	# Inputs	# TOs	COMIX		MIO		vMOSA		Single	
			Mean	S.d	Mean	S.d	Mean	S.d	Mean	S.d
SBANK	1	5	**0.9977**	0.0005	0.9509	0.0600	0.9934	0.0010	0.8452	0.1869
	2	5	**0.9901**	0.0125	0.8335	0.1054	0.9544	0.0679	0.1197	0.1239
	3	5	**0.9364**	0.1241	0.5861	0.1602	0.8477	0.1158	0.0133	0.0506
SSBANK	1	5	**0.9944**	0.0010	0.9909	0.0015	0.9913	0.00085	0.8168	0.1844
	2	5	**0.9825**	0.0165	0.4594	0.3091	0.9773	0.03548	0.0066	0.0363
	3	5	0.6973	0.2971	0.0090	0.0356	**0.7080**	0.2808	0.0000	−
XMLMAO	1	5	0.9230	0.0211	0.8400	0.0650	**0.9550**	0.0097	0.7925	0.1764
SBANK	1	50	0.9216	0.0082	0.7264	0.0629	**0.9298**	0.0316	0.6845	0.0535
	2	50	0.7358	0.0406	0.4334	0.0464	**0.8013**	0.0571	0.1288	0.0484
	3	50	**0.8979**	0.0464	0.2508	0.1039	0.8607	0.0443	0.0302	0.0218
SSBANK	1	50	**0.7724**	0.0624	0.7236	0.0240	0.7511	0.1597	0.6800	0.0875
	2	50	**0.2535**	0.1641	0.1892	0.0885	0.1826	0.2176	0.0019	0.0074
	3	50	**0.2564**	0.3413	0.0618	0.0995	0.2035	0.3324	0.0000	−
XMLMAO	1	50	0.74924	0.0508	0.5642	0.0992	**0.7533**	0.0524	0.3947	0.0839
M	2	50	**0.0020**	0.0038	0.0154	0.0044	**0.0020**	0.0060	0.0000	0.0000

According to Table 1, with the edit distance as fitness function, COMIX achieves 100% of coverage most of the time when optimizing only five TOs. For the larger set of TOs, its coverage ranges between 21% and 100%. Instead, MIO and vMOSA are very competitive only when dealing with five TOs: the coverage obtained by vMOSA ranges between 75% and 100% while for MIO it ranges between 80% and 100%. However, when the goal is to optimize 50 TOs, MIO and vMOSA yield zero coverage in most of the cases. The only exception is XMLMAO, for which vMOSA and MIO achieve 38% and 61% coverage, respectively; for the same subject, COMIX reaches a coverage of 100%. The single-target algorithm turns out to be the worst search strategy: it achieved a low coverage (≤10%) for SSBANK with two and three inputs even when targeting only five TOs. Similar to MIO and vMOSA, the single-target algorithm often yields zero coverage when optimizing the largest set of TOs.

For those cases where COMIX achieves the same level of coverage as MIO and vMOSA, we compare the corresponding AUC values, as reported in Table 3. As we can observe from the table, COMIX achieved higher AUC values for all cases where coverage results were similar to other algorithms. For example, all algorithms achieved 100% coverage for SBANK with 1 input, but the AUC value for COMIX is higher. This means that, for this subject, our co-evolutionary algorithm was able to cover all TOs in SBANK in less time compared to the alternative algorithms.

Coverage results of the algorithms when using the linear distance are shown in Table 2.

With this fitness function, the algorithms achieved 90-100% coverage in 11 (COMIX), 9 (vMOSA) and 7 (MIO) case study settings out of 16.

The corresponding AUC values are also in accordance with the coverage results, as reported in Table 4. For the smaller set of five TOs, vMOSA achieved higher AUC values than COMIX in 1 out of 7 experiments. For 50 TOs, COMIX exhibited the highest AUC values in most of the experiments. Similar to the results with edit distance, the single-target algorithm was found to be the worst when using the linear distance.

**Fig. 3:**
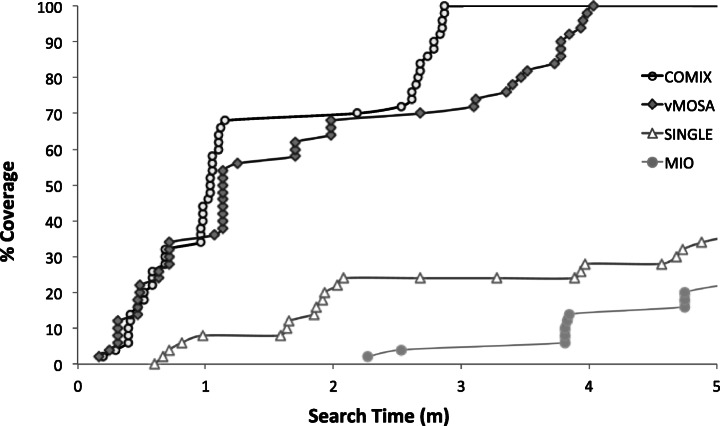
% Coverage achieved by different algorithms when using linear distance, for SBANK with one-input and 50 TOs

Figure 3 depicts the coverage obtained (during the first five minutes of the search) by the four algorithms when using linear distance, for the experiment with the one-input version of SBANK when optimizing 50 TOs. As shown in the figure, COMIX exhibited the best performance by achieving 100% coverage in less than three minutes, while vMOSA needed more than four minutes to reach the same level of coverage. In contrast, the single-target search and MIO could only achieve less than 40% coverage within five minutes. As a result, COMIX has the highest AUC value among all alternatives.

*Statistical analysis*.: According to the Friedman’s test, the various combinations of distances and algorithms have statistically different AUC values (*p*-value = 2.18^− 5^) with 50 TOs. To help understand which are the best combinations, the final ranking produced by the Friedman’s test is reported in Table 5. The results of the pairwise comparison (the pairwise Wilcoxon test) are also reported in Table 5. As we can notice, COMIX with linear distance is ranked first and is significantly better than all other combinations in the comparison. vMOSA (the MOSA variant customized for *XMLi*) with linear distance is ranked second and statistically outperforms all other combinations. Finally, we notice that MIO with linear distance is ranked third but it is not statistically better then COMIX with edit distance, which is ranked fourth.

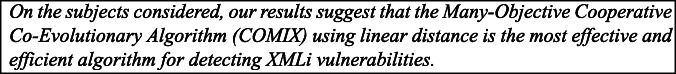


**Table 5:** Ranking produced by the Friedman’s (smaller values of Rank indicate better AUC values) and statistical significance by the pairwise Wilcoxon test

ID	Algorithms	Rank	Significantly better than
(1)	COMIX-Lin	1.88	(2), (3), (4), (5), (6), (7), (8)
(2)	vMOSA-Lin	2.25	(3), (4), (5), (6), (7), (8)
(3)	MIO-Lin	3.50	(5), (6), (7), (8)
(4)	COMIX-Ed	3.75	(6), (7), (8)
(5)	MIO-Ed	5.31	(8)
(6)	SINGLE-Lin	5.63	−
(7)	vMOSA-Ed	6.81	−
(8)	SINGLE-Ed	6.88	−

### RQ2: Is the Execution Time to Achieve Maximum Coverage for a Given Set of TOs Acceptable in Practice?

Table 6 reports the execution time required to achieve the maximum coverage by COMIX with linear distance, which is the most efficient and effective strategy according to the results of **RQ1**. For this analysis, we focus only on 50 TOs as, in practice, security analysts are interested in discovering as many *XMLi* vulnerabilities as possible within minimum time. As we can observe from the table, the execution time ranges between 3 and 23 minutes for SBANK, SSBANK, and XMLMAO. The maximum running time is that of SSBANK with three inputs. For the industrial case study (e.g., *M*) the running time is up to 175 minutes (e.g., less than three hours). Such a larger running time is because test cases in *M* are more expensive to run compared to the other systems. Indeed, in SBANK, SSBANK, and XMLMAO, one single test case execution corresponds to 1-2 milliseconds on average compared to 400ms spent on one single test execution in *M*, on average. Based on our experience, finding *XMLi* vulnerabilities in web-applications in (at most) few hours is reasonable in practice as the vulnerability analysis can be run overnight.

**Table 6:** Average time (in minutes) required to reach the maximum coverage for 50 TOs when executing COMIX with linear distance

System	SBANK			SSBANK			XMLMAO	M
# Inputs	1	2	3	1	2	3	1	2
Time	3.83	15.44	5.84	9.04	21.37	22.41	19.96	175.87

Therefore, for **RQ2**, we conclude that: 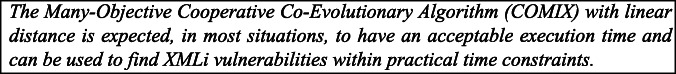


### RQ3: What is the Impact of the Linear Distance on the Fitness Calculation Time?

To better understand the impact of the fitness function on the running time of COMIX, Table 7 reports the string distance calculation time when using edit distance (FC_ed_) and linear distance (FC_lin_), for the set of 50 TOs.

**Table 7:** Fitness calculation times (% of total execution time) for COMIX with 50 TOs when using Edit Distance (FC_ed_) and Linear Distance (FC_lin_)

System	# Inputs	FC_ed_	FC_lin_
SBANK	1	85.10	0.59
	2	85.07	0.84
	3	95.26	1.34
SSBANK	1	80.15	0.64
	2	73.43	0.43
	3	92.88	1.97
XMLMAO	1	14.03	0.04
M	1	1.04	0.00093

As we can observe from the table, the distance calculations for the edit distance are very expensive compared to linear distance. For instance, for SBANK with one input, COMIX spent 84.33% of the total execution time on the string distance calculations. On the other hand, when using the linear distance, the distance calculations took less than 1% of the total execution time. Similar differences in the distance calculation times can be observed for the other applications.

However, the impact of distance calculations is strongly related to the complexity of the case study. For example, although the edit distance is roughly 1,000 times slower than the linear distance on *M*, such cost is only 1% of the fitness evaluation. The more complex an application is, the less impact the choice of distance will be on performance.

To summarize, the edit distance is more expensive to compute and can consume most of the search budget because of its higher computational complexity, i.e., $\mathcal {O}(n \times m)$, as opposed to linear distance with its linear time

complexity $\mathcal {O}(n)$. While the linear distance may provide less search guidance than the edit distance, its low computation time is a major advantage in terms of search effectiveness as it can enable the execution of many more COMIX generations within the same time.

Therefore, for **RQ3**, we can conclude that: 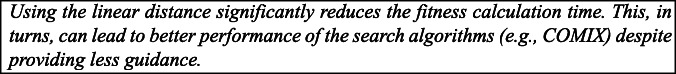


### The Impact of the Migration Rate on the Performance of COMIX

As described in Section 2.3, in co-evolutionary algorithms, the strongest individual of the winning island is migrated to others islands to improve genetic diversity. However, in our context, the TOs are different and independent from each other: if one test case covers one TO, it cannot cover other TOs at the same time. This specificity may render the migration ineffective.

In our empirical study, the migration rate was set to one single test case selected from the island that wins the migration.

To assess whether the migration policy impacts the performance of COMIX, we ran our algorithm with different *migration rates*. Table 8 reports the TO coverage achieved by running COMIX when varying the number of migrated tests from zero (no migration) to 50 (i.e., all test cases are migrated to different islands). For the sake of analysis, we focus on SBANK with three test inputs and use the linear fitness function. The leftmost column in the table reports the number of migrated individuals while the second and third columns report the percentage of TOs covered within two and five minutes of execution, respectively. Since each experiment was repeated 10 times to account for the randomized nature of the algorithm, we report the average values for TO coverage.

**Table 8:** TO Coverage (%) achieved with different migration percentages when using COMIX with linear distance for SBANK

# Migrated	% Covered TOs	
Tests	in 2 mins	in 5 mins
0 (0%)	11.43	29.60
1 (2%)	53.60	93.11
2 (4%)	46.80	95.77
3 (6%)	47.40	94.40
5 (10%)	33.20	89.20
10 (20%)	35.20	81.80
50 (100%)	7.60	45.60

As we can observe from the table, coverage is very low in the absence of migration: less than 12% and 30% of the TOs are covered within the first two and five minutes, respectively.

Instead, when the migration rate is increased from 0 to 10%, a drastic increase in coverage can be observed within the same execution time, i.e., from 29.60% to 93%-95% for five minutes. However, further increases in migration rate, from 10% to 100%, lead to a lower number of covered TOs within the same amount of time. This trend in coverage is due to the increased overhead of the migration policy: every time a test case *t* is migrated from the source island to the target ones, *t* is re-evaluated to compute the distance function to cover the corresponding TOs. When the migration rate is 100%, then all test cases are migrated and evaluated against all TOs, similarly to vMOSA and MIO.

Finally, from a statistical point of view (using the Wilcoxon test), COMIX with the setting used in our empirical study (e.g., one test case migrated per iteration) achieves a significantly higher coverage than all other settings with two minutes of search budget (all *p*-values are <0.01). However, when the search time is set to five minutes, there is no statistically significant difference when varying the migration rate from 2% to 10%, though zero or rates higher than 10% still lead to significantly lower coverage.

## Threats to Validity

Threats to internal validity come from the fact that our empirical study is based on a software prototype. We implemented different search algorithms, and possible differences in performance might be due to bugs or inefficiencies in their implementation details.

We carefully tested our implementations, but we cannot guarantee that they are bug-free.

The fact that a web application can be led to send malicious messages to internal web services does not necessarily mean that such web services will be compromised. It depends on how such service will process these messages. As a result, the number of found TOs is only an upper bound to the number of discovered vulnerabilities that can be exploited.

In any case, it is still safer if this kind of malicious messages are never sent, as bugs in new releases of these internal web services could lead to security breaches.

Regarding conclusion validity, our study is based on randomized search algorithms, which exhibit some degree of random variation in their results. Therefore, each experiment was repeated 30 times (10 for the industrial system), and the resulting data were analyzed with appropriate statistical tests, like for example the Friedman’s test (García et al. 2008).

Threats to external validity come from the fact that any feasible empirical study on such a topic is necessarily limited to a small number of systems and inputs, mostly given the substantial computational time required to run our experiments (about 800 hours). In our case, we rely on three open source systems and an industrial one. More case studies are required to be able to better generalize the findings of this paper.

However, as the used industrial system is a very typical enterprise application, we can expect that our novel technique could be successful with other similar systems.

## Related Work

In this section, we describe work related to testing techniques for vulnerability detection in web applications. We also discuss search-based testing and our previous work on *XMLi* (Jan et al. 2017a, 2017b) that we extend in this paper.

### Security Testing of Web Applications/Services

Security testing techniques of web applications can be divided into two main categories: based either on (i) White-box testing or (ii) Black-box testing.

***White-box testing***: In White-box testing techniques, information about the internal workings of the SUT (web application) is available to the tester, e.g., source code, bytecode and/or design documentation. Such information is used to generate test inputs (attacks) to assess the security of the web application.

Several white-box testing techniques (Livshits and Lam 2005) have been proposed in the literature (Jovanovic et al. 2006b; Halfond et al. 2008; Kieyzun et al. 2009; Jovanovic et al. 2006a; Chess and West 2007; Huang et al. 2004; Livshits and Lam 2005) for the detection of web application vulnerabilities, e.g., SQL Injection and Cross-site Scripting. One of such white-box security testing techniques is “taint” analysis (Jovanovic et al. 2006b; Wassermann and Su 2007), which is used to identify vulnerable execution paths by statically detecting the data coming from untrusted (tainted) sources. Halfond et al. (2006, 2008) proposed a taint analysis based approach and a tool, namely WASP, for protecting web applications against SQL Injection attacks. Their approach identifies trusted data sources, use dynamic tainting to track trusted data at runtime, and allow only trusted data to be used in SQL queries. Clause and Orso (2009) also proposed an approach and tool, Penumbra, based on dynamic tainting. Penumbra identifies failure-relevant inputs from a given set of failure-inducing inputs and an observable faulty behavior of the SUT. Avancini and Ceccato (Avancini and Ceccato 2010) have also proposed an approach to improve taint analysis by integrating with genetic algorithms for detecting cross-site scripting vulnerabilities in web applications. Their approach first identifies the vulnerable execution paths via taint analysis, and then use genetic algorithms to make the execution flow traverse the identified target paths. Another white-box testing approach based on static analysis and runtime protection is proposed by Huang et al. (2004). Their approach uses Type-based (Strom and Yemini 1986) and data-flow analysis (Allen and Cocke 1976) to identify vulnerable parts of the code (those using untrusted data) and inserts sanitization routines there.

All of the above white-box testing approaches require access to the source code of the web application and may need to modify it (e.g., by doing code instrumentation) of the web application. At times, this might not be feasible in practice, e.g.,

when the security testers are not the developers of the application. Even in the presence of source code, such techniques can only work with known attack patterns that might become out-dated. Dynamic code analyses have also intrinsic limitations due to their complexity, e.g. tools like WASP do not handle “primitive types, native methods, and reflection” (Halfond et al. 2008). And a white-box testing tool is limited only to the specific type of language it supports, e.g., a tool targeting Java will not be able to handle all the other popular languages used in web/enterprise development such as C#, PHP, JavaScript, Python, Ruby on Rails. This is a particular problem considering current trends in industry, where different languages are often used together in the same *microservice* architecture. Moreover, none of these techniques target *XMLi* vulnerabilities.

In contrast, COMIX and our baselines are black-box security testing techniques targeting *XMLi*. They do not rely on source code and search for unknown inputs that can detect *XMLi* in the SUT. They can be applied to any type of language in which the web applications are written (e.g., in our case study, both PHP and Java were used).

***Black-box testing:*** Black-box security testing techniques are widely used in scenarios where no insights about the internal working (e.g., source code) of the application are provided to the tester. There is a large research body investigating such techniques for the detection of web application/services vulnerabilities, e.g., Huang et al. (2005), Mainka et al. (2012), Chunlei et al. (2014), Chen et al. (2014), and Kieyzun et al. (2009). A common issue with most of these approaches is the large number of false positives, which makes their application in practice difficult.

Bau et al. (2010) performed a study to evaluate the effectiveness of the state-of-the-art in automated vulnerability testing of web applications. Their results demonstrate that such approaches are only good at detecting straightforward historical vulnerabilities but there exist more room for research in detecting advanced forms of vulnerabilities and lowering the false positive rates of the current state-of-the-art. Besides, none of these approaches are dedicated to the detection of XML injections, the objective of this paper.

In the following section, we discuss existing literature on *XMLi* vulnerabilities and techniques that are closely related to our work, i.e., search-based testing.

### Testing for XML Injections

Unlike SQL injection and cross-site scripting vulnerabilities that received much attention (e.g., Appelt et al. 2014, 2015; Gallagher 2008; Junjin 2009), only limited research targets XML injections. An approach for the detection of XML injection attacks is presented by Rosa et al. (2013). They proposed a strategy to first build a knowledge database from the known attack patterns and then use it for detecting XML injection attacks, when they occur. This approach is an improvement over the traditional signature-based detection approaches but it focuses on intrusion detection and not on security testing. In contrast, our work targets test data generation to detect XML injection vulnerabilities in web applications.

A basic testing methodology for XML injections is defined by OWASP (Testing for XML Injection 2016). It suggests to first discover the structure of the XML by inserting meta-characters in the SUT. The revealed information, if any, combined with XML data/tags can then be used to manipulate the structure or business logic of the application or web service. OWASP also provided a tool named WSFUZZER (WSFuzzer Tool 2016) for SOAP penetration testing with fuzzing features. However, as reported in Jan et al. (2016), the tool could not be used with WSDLs having a complex structure (nested XML elements) and is only useful in scenarios where the web services are directly accessible for testing.

In our previous work (Jan et al. 2016), we discussed four types of XML injection attacks and proposed a novel approach for testing web services against these attacks. Our evaluation found the approach very effective compared to state-of-the-art tools. However, it focuses on the back-end web services that consume XML messages and are directly accessible for testing. In contrast, our current work targets the front-ends (web applications) of SOA systems that produce XML messages for web services or other back-end systems.

In addition, while in Jan et al. (2016) we used constraint solving and input mutation for manipulating XML messages, in this paper we use search-based testing techniques to generate test inputs for the front-end of the SUT that produces malicious XML messages. Such inputs can then help detect XMLi vulnerabilities in web applications that can be exploited through the front-ends.

### Search-Based Approaches for Security Testing

Search-based software testing has mostly focused on functional testing (Fraser and Arcuri 2014; Harman 2007; McMinn 2004b; Harman and McMinn 2010) while non-functional aspects, and especially security testing, have received only limited attention (Afzal et al. 2009; Türpe 2011). Avancini and Ceccato (2011) have used search-based testing for detecting cross-site scripting vulnerabilities in web applications. First, they use static analysis to detect candidate cross-site scripting vulnerabilities in PHP code. A genetic algorithm together with a constraint solver is then used to search for input values that can trigger the vulnerabilities. In contrast, our approach is a black-box testing technique that targets *XMLi* vulnerabilities.

Thomé et al. (2014) also used a search-based technique for the security testing of web applications. Their approach systematically evolves inputs to expose SQL injection vulnerabilities by assessing the effects on SQL interactions between the web server and database. Our search-based testing approach also focuses on evolving test inputs but we address a different type of vulnerabilities, *XMLi* attacks. Moreover, Thomé et al. used a fitness function based on a number of factors that indicate the likelihood that the output is resulting from *SQLi* attacks. In contrast, we use a fitness function based on the distance between the SUT’s outputs and automatically derive test objectives based on attack patterns.

There exist other vulnerability detection techniques (Del Grosso et al. 2008; Rawat and Mounier 2011) that rely on evolutionary algorithms. Unlike our black-box approach for *XMLi* testing, these techniques are white-box and are used for buffer overflow detection.

To the best of our knowledge, search-based testing has never been used for the detection of *XMLi* vulnerabilities in web applications that deliver XML messages to corporate web services.

#### Previous Work and Current Extension

In our previous work (Jan et al. 2017a), we presented a search-based approach for generating test inputs exploiting XML injection vulnerabilities in front-end web applications. We used the standard Genetic Algorithm (*SGA*) along with the string-edit distance (*Ed*) to find malicious test inputs. We evaluated our approach on several web applications including a large industrial application and we also compared it with random search. We found our proposed search-based testing approach to be very effective, as it was able to cover vulnerabilities in all case studies while the random search could not, in any single case. We further extended this work in Jan et al. (2017b) by investigating two additional optimization algorithms, namely Real-coded Genetic Algorithm (*RGA*) and Hill Climbing (*HC*). We also introduced a different fitness function i.e., the Real-coded Edit Distance (*Rd*), which further improves the traditional string edit distance (*Ed*). Our empirical evaluation showed that *RGA* with *Rd* is significantly superior to the previous approach (Jan et al. 2017a) in terms of both effectiveness and efficiency.

Both of our previous works (Jan et al. 2017a, 2017b) are based on single-target search-based techniques (i.e., searching for each malicious message independently), which may face scalability challenges with large applications where the search is required for many potential malicious messages to uncover multiple vulnerabilities at the same time. The current paper extends our previous works in several ways. First, we proposed a novel co-evolutionary testing technique, namely COMIX. Second, we investigated and adapted the two multi-target search techniques, namely MOSA and MIO, which are the two most recent multi-target white-box unit testing techniques. Finally, we investigated the usage of an alternative fitness function, which is less expensive than our previously used fitness functions (*Ed* and *Rd*). Our results show that the multi-target techniques outperform the single-target and our novel technique COMIX, when used with our proposed fitness function, is significantly more effective and efficient than all investigated alternatives.

## Conclusion and Future Work

Security testing of the front-ends of enterprise systems is crucial for their overall security. Such front-ends are the first point of contact with the user. For example, if they are vulnerable to XML Injections (*XMLi*), then they can be tricked to generate and send malicious XML messages to internal services (e.g., SOAP web services).

And though there exist testing techniques that can possibly lead to the generation of malicious, potentially harmful XML messages, these techniques target each malicious XML message one at a time. Therefore, they are inefficient when testing the security of larger web applications that require testing for many potential XML messages, especially in the presence of strict input validation/sanitization routines and time constraints.

In this paper, we have presented a novel co-evolutionary testing technique, namely COMIX, to address the scalability challenges of the existing single-target approach for *XMLi*. Moreover, as baselines of comparison, we have investigated and adapted the two most recent multi-target, white-box unit testing techniques, namely MOSA and MIO, to *XMLi* testing. Last, we have proposed and evaluated an alternative fitness function, which is less expensive than the string edit distance used in the literature to guide the search for matching strings.

We have carried out an experimental evaluation to compare our proposed co-evolutionary algorithm (COMIX) and fitness function with existing approaches. Our subjects for evaluation include: (i) six different variants of a front-end web application for a real-world bank card processing system, (ii) one open-source web application vulnerable to *XMLi*, and (iii) one large real-world industrial application.

Consistent with our expectations, our case study results provide empirical evidence that COMIX, when combined with our proposed fitness function, is significantly more effective and efficient in finding *XMLi* vulnerabilities, when compared to other combinations of search algorithms and fitness functions, including both multi-target and single-target techniques. Though more studies are of course required to confirm our results, COMIX is not based on any assumption that is particularly advantageous for our case studies.

COMIX is not limited to XML Injections. It is a generalizable approach and can be adapted to test web applications for other types of attacks. To do so, one only needs to modify the Test Objectives (TOs) according to the corresponding types of attacks. In our context, TOs are malicious XML messages which are essentially strings for the proposed search technique (COMIX). For other types of attacks, only such messages (strings) need to be modified and no changes to the implementation of the search technique are required.

In addition to XML, many systems now use the JSON format for data exchange. There are two options to apply COMIX to such systems: (1) modify the existing TOs by inserting malicious content in JSON messages, (2) if the system also supports XML, convert the JSON inputs to XML and use the same set of TOs. The latter option can easily be integrated into COMIX as there exist many tools/plugins for converting JSON to XML and vice versa. In either case, once again, the implementation of the search technique will not require any modifications.

Our future work will extend the current approach to cover more vulnerabilities and data exchange formats.

## Footnotes

1 The name of the company cannot be revealed due to a non-disclosure agreement

2 https://github.com/SpiderLabs/MCIR

3 7 *a**p**p*. *v**e**r**s**i**o**n**s* × 4 *a**l**g**o**r**i**t**h**m**s* × 30 *m**i**n**u**t**e**s* × 30 *r**e**p**e**t**i**t**i**o**n**s* = 25200 *m**i**n**u**t**e**s**o**r* 420 *h**o**u**r**s*
